# Reliability analysis of a novel measurement system for quantifying human skin color

**DOI:** 10.1002/ski2.182

**Published:** 2022-10-17

**Authors:** Chien‐Chung Chen, Cheng‐Yin Chung, Yi‐Wen Chiu, Yu‐Hsuan Lin, Ling‐Shan Tse, Ching‐Ying Wu, Shang‐Jyh Hwang, Ming‐Yen Lin, for the iH research group

**Affiliations:** ^1^ National Taiwan University Hospital National Taiwan University Taipei Taiwan; ^2^ Department of Internal Medicine Division of Nephrology Ministry of Health and Welfare Pingtung Hospital Pingtung Taiwan; ^3^ Department of Internal Medicine Division of Nephrology Kaohsiung Medical University Hospital Kaohsiung Medical University Kaohsiung Taiwan; ^4^ Department of Renal Care College of Medicine Kaohsiung Medical University Kaohsiung Taiwan; ^5^ Taiwan Instrument Research Institute National Applied Research Laboratories Hsinchu Taiwan; ^6^ Medical Science Liaison WS Far IR Medical Technology CO., LTD Taipei Taiwan; ^7^ Department of Dermatology Kaohsiung Municipal Ta‐Tung Hospital Kaohsiung Taiwan

## Abstract

**Background:**

Precision is crucial in determining the appropriate procedure for implementing further trials. We conducted a study to explore the reliability of a novel measuring system for human skin color.

**Methods:**

The novel skin color measuring system was used to capture the skin color of four volunteers (2 males and 2 females) from the same location on each subject by the same operator. The measurement was repeated for different poses and instrument factors (camera and shooting protocol) in the red, green, and blue (RGB) system. The average color depth in each image was calculated and converted from 0 to 255. The spread of measures and the Bland‐Altman plot was displayed to determine each variance source's random error, with the interclass correlation coefficients applied to reflect the reliability.

**Result:**

The RGB color depth in the experiment ranged from 190, 152, and 122 to 208, 170, and 142. The 95% confidential interval of the differences from the means in RGB colors for the different protocols were ±2.8, ±2.6, and ±2.1, respectively. The largest variation in the replicate trials was observed when subjects were in a supine position (standard deviation: 2). The interclass correlation coefficients were greater than 90%, suggesting that the developed system is highly precise.

**Conclusion:**

This study demonstrated that the developed device could stably and reliably detect human skin color across different common sources of variation, and thus could be applied clinically to explore relationships between health/disease and skin color changes.

## Graphical abstract

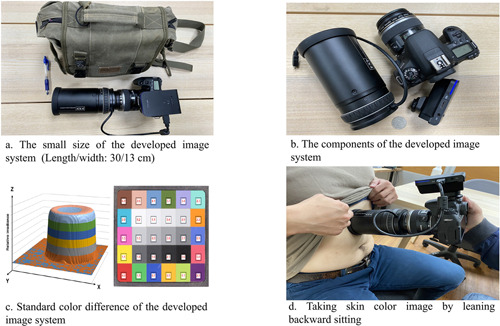

To practically quantify human skin health, we developed a highly accurate, portable, and low‐cost skin color image system. The current study shows a high consistency measurements regardless of different cameras, shot models, and subject postures by this developed skin color image system. The system is worth applying clinically to further explore the relationships between health/disease and skin color changes.

1

**What is already known about this topic?**

- Several instruments have been recently employed to estimate human skin color for relevant research, however, these devices mainly adopted point imaging systems at extreme costs, making them less practical to apply in practice.

**What does this study add?**

- This study reports the highly accurate, portable, and low‐cost skin color image system testing on four subjects and confirms its reliability regarding the cameras, shot model, and subject posture. The 95% confidential interval of the differences from means in RGB colors for different protocols are ±2.8, ±2.6, and ±2.1, respectively. The interclass correlation coefficients are over 90%, suggesting the developed system is highly precise.

## 2 INTRODUCTION

The skin is the largest organ of the human body composing 18% of body weight which protects the body from external injuries. It also helps regulate body temperature, the pH of bodily fluids, and excrete substances, maintaining a weak acid environment to prevent microbial infection.^1^, ^2^ Several physical features of the skin may reflect specific health problems such as color, temperature, elasticity, dryness, fine wrinkling, atrophy, and laxity. Developing an accurate and precise instrument to quantity physical features of skin could be helpful for health promotion and disease prevention.^3^, ^4^, ^5^

Skin color is influenced by several factors such as ethnicity, lifestyle, or even biochemical. It also partly reflects specific social classes that are engaged in more outside activities.^6^ In addition, skin color changes are potentially an indicator of disease. For example, hyperpigmentation (skin color turning to black) is an important differential diagnosis for Addison's disease,^7^ whereas jaundice (skin color turning to yellow) may also be a symptom or sign to identify patients with alcoholic hepatitis.^8^ Determining skin color change by the human eye is usually subjective, ambiguous depends on experience, uncertainty, and not reproducible, therefore an instrument to quantify skin color would be helpful in a range of relevant fields.

Several instruments have been recently employed to estimate human skin color but these devices are mainly adopted point imaging systems which are costly and not practical to apply in practice.^9^ We previously reported that the developed skin color system could accurately quantify human skin color,^10^ so this study aimed to quantify the reliability of the developed skin color system for catching images from the abdomen which is less influenced by sun exposure.

## 3 MATERIALS AND METHODS

### 3.1 Imaging system for skin color

**FIGURE 1:**
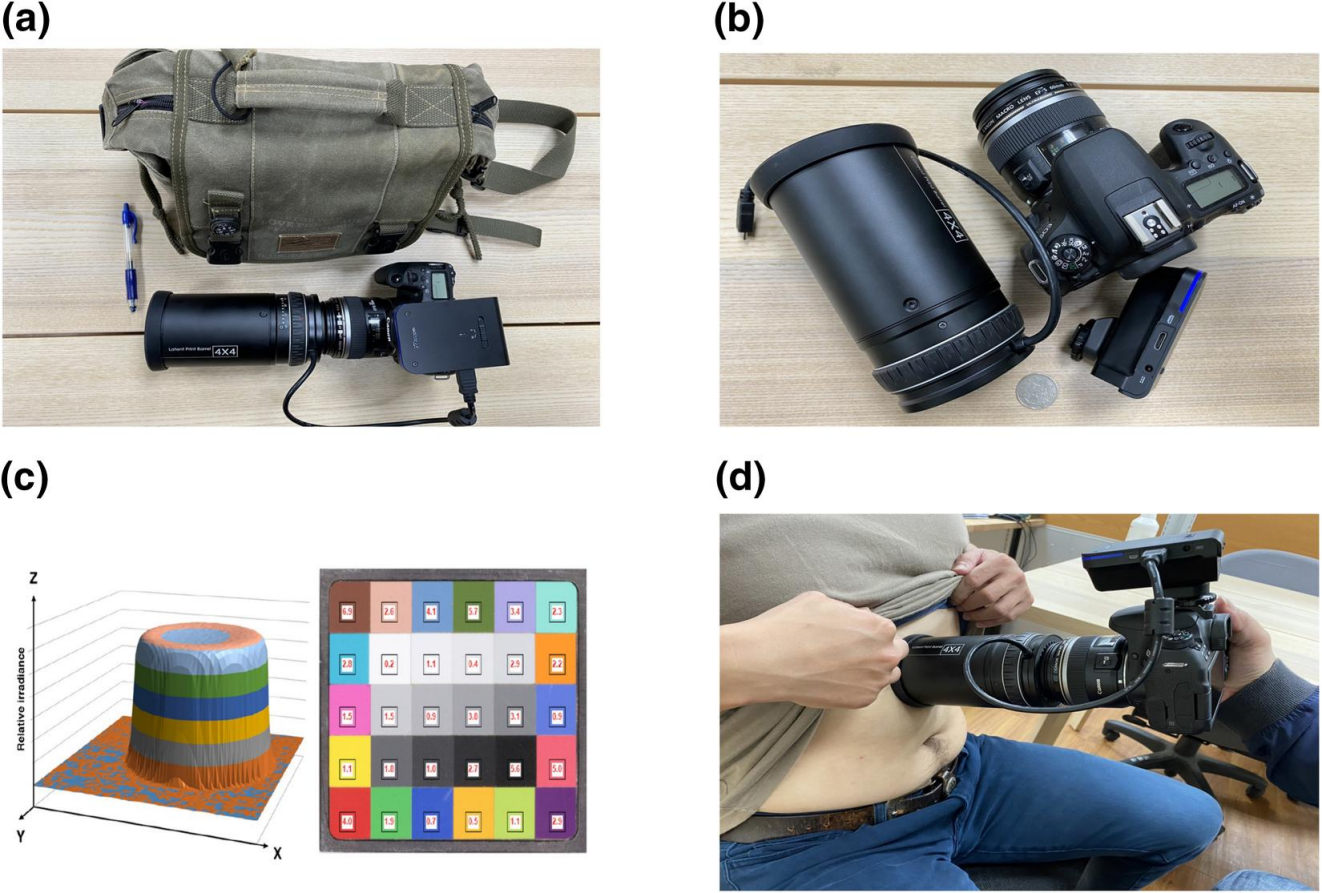
The highly accurate, portable, low‐cost skin color image system. The system weighs 1.5 kg and is 30 cm in length and 13 cm in width. It composites three key elements: a sunlight light‐emitting diode ring light, a dark room module, and a digital single‐lens reflex camera (Canon DSLR, 77D) with a macro lens (EF‐S, 60 mm). The plot of the standard color difference was derived from our previous conference paper.^10^ The 1d) exhibits that applying the imaging system takes a leaning backward sitting subject's skin color image. (a) The small size of the developed image system (length/width: 30/13 cm). (b) The components of the developed image. (c) Standard color difference of the developed image system. (d) Taking skin color image by leaning backward sitting.

The imaging system was co‐developed by the Taiwan Instrument Research Institute and Kaohsiung Medical University Hospital. In brief, the imaging system was composed of three major components, a sunlight light‐emitting diode ring light, a dark room module, and a digital single‐lens reflex camera (Canon DSLR, 77D) with a macro lens (EF‐S, 60 mm). The system can catch standardized color through the controlled light source and a precise light censoring module. The newly developed device quantified colors by the Red‐Green‐Blue (RGB) color system that are easily transformed to *l*, *a*, *b* color system (Figure 1). Previously, we confirmed that the system could detect the color difference that the typical human eye could not identify. To ensure the stability of the color system and optimal operational procedure for catching human skin color, we quantified four main variances of colors: cameras, people, subject poses, and acquisition procedures. All of the study procedures were approved by the Institutional Review Board of Kaohsiung Medical University Hospital (KMUHIRBEXEMPT(I)‐20170144).

### 3.2 Subjects and measurements

The healthy volunteers (2 men and 2 women with an average age of 38 years; range: 26–48) were randomly selected from the Kaohsiung Medical University Hospital located in southern Taiwan (22°′N 120°′E). Their jobs were mainly indoor so they were not excessively exposed to solar radiation and they reported that they had not been exposed to excessive sunlight 3 months before the experiment. One experienced research assistant performed the experimental procedures in a room without outside interferences in spring 2018. The skin images were all taken from the abdominal skin 10 cm under the central area of the umbilicus (Figure 1). In order to avoid interference from other light sources, the lens gently covers the skin when taking photos. The images were collected from the same body area with three cameras (I, II, III) according to (1) three shooting protocols:

(A) 10 continuous shots
(B) 3 shots without picking up the camera
(C) 3 shots with a camera picked up between each shot;

and (2) four postures of observation:

(a) supine
(b) leaning backward sitting
(c) slumped sitting
(d) standing

### 3.3 Data processing and analysis

The instrument RGB system displayed human skin color, which contains three primary colors by 0–255 color depths. We calculated the average color depth ($\bar{CD}$) of each primary color using the following formula:

$${\bar{CD}}_{\left\{R,G,B\right\}}=\frac{\sum_{c=0}^{255}Color\;depth*Pixels}{Total\;pixel\;number\;in\;one\;picture}$$

The average color depth was obtained for each skin image, and the range of average color depth was described by plots to reflect the maximum changes in skin color measurements in all experimental procedures (human, camera, protocol, and postures). We classified pictures shot in the same camera, posture, and protocols to the same group. The averages and the differences from the average within protocol and postures were calculated and displayed by Bland‐Altman plots.^11^ The measurements outside the 95% confidence intervals (CIs) of the differences from the average were considered outliners. The number of outliners and dispersions of the differences from the average color depth in each RGB color were evaluated to reflect the consistency between experiments. To understand the stability of our skin color detecting device under different groups, cameras, shooting protocols, and postures, interclass correlation coefficients (ICC) and 95% CIs were displayed. All statistical analyses were performed using SPSS statistical software (version 25; IBM Corp.).

## 4 RESULTS

### 4.1 Skin color measurements

**FIGURE 2:**
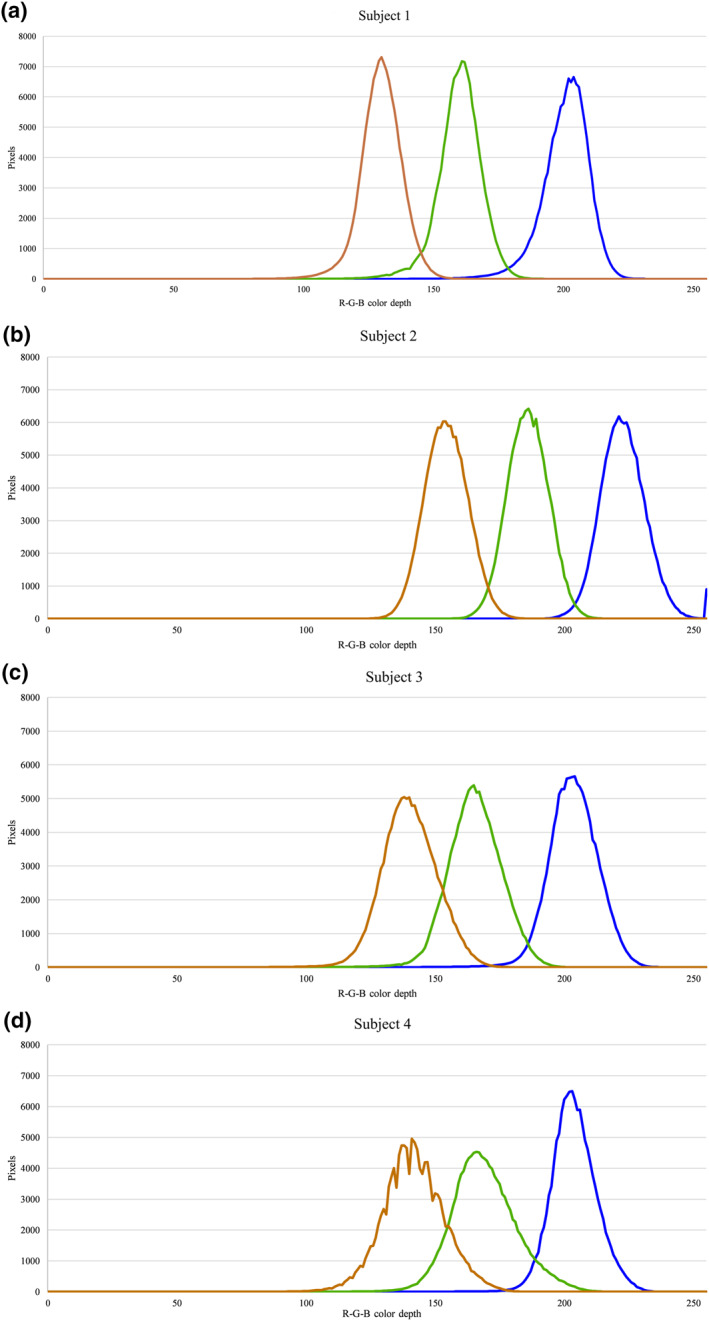
The distribution of skin color depth in the red‐blue‐green colors of (a) subject 1, (b) subject 2, (c) subject 3, and (d) subject 4. Each color depth of the red‐blue‐green colors was the average of repeated experimental values captured by the developed image system.

Our trial took 768 skin color pictures classified by different cameras, protocols, and postures. The distribution of the RGB color spaces quantified by the developed image system from subjects' skin was similar. The minimum average color depth in the RGB colors of the four subjects was 190, 152, and 122, whereas the maximum was 235, 196, and 165, respectively (Figure 2). The Bland‐Altman plots for the repeated measurements of the different shooting protocols (displayed by a different color) are shown in the supplement. Most average color depths in all Bland‐Altman plots were clustered in lower values, suggesting that one subject's skin color may be lighter than others (Figure S1a–c). The parameters produced from Bland‐Altman plot analyses based on the combination of shooting protocols are displayed in Table 1. A similar average color depth in RGB colors (208, 170, 142) was found in both shooting protocols A (10 continuous shots) and B (3 shots without picking up the camera) but was smaller for the 2 color depth for shooting protocol C (3 shots with camera picked up between each shot). All standard deviations of the difference from the average of each color space were less than 1.5, and the 95% CI of the differences was less than 3 in line with our previous reported error (∆E < 3).

**TABLE 1:** Parameters of the Bland‐Altman plot for color depth comparison of the three protocols

		Difference from average in color depth			Average in color depth		
Measurements		Red	Green	Blue	Red	Green	Blue
Protocol A, B, and C^a^ (*n* = 432)	Mean	0.0	0.0	0.0	207.8	169.7	141.0
	Limits of agreement	±2.8	±2.6	±2.1			
	SD	1.4	1.3	1.1			
	No. of outlier (H/L)	12/13	14/10	13/10			
Protocol A and B (*n* = 288)	Mean	0	0	0	208.3	170.4	141.7
	Limits of agreement	± 2.6	± 2.1	± 1.8			
	SD	1.3	1.1	0.9			
	No. of outlier (H/L)	4/6	7/10	6/8			
By protocol B and C (*n* = 288)	Mean	0	0	0	207.5	169.2	140.4
	Limits of agreement	±2.8	±2.6	±2.1			
	SD	1.4	1.3	1.1			
	No. of outlier (H/L)	10/11	10/7	9/7			
Protocol A (*n* = 144)	Mean	0	0	0	208.5	170.7	142.2
	Limits of agreement	± 2.6	± 2.3	± 1.9			
	SD	1.3	1.2	1.0			
	No. of outlier (H/L)	10/10	15/14	14/11			
Protocol B (*n* = 144)	Mean	0	0	0	208.2	170.0	141.3
	Limits of agreement	± 2.4	± 2.1	± 1.6			
	SD	1.2	1.1	0.8			
	No. of outlier (H/L)	4/6	2/6	2/5			
Protocol C (*n* = 144)	Mean	0	0	0	206.8	168.4	139.4
	Limits of agreement	± 3.1	± 3.1	± 2.5			
	SD	1.6	1.3	1.3			
	No. of outlier (H/L)	8/10	5/3	5/3			

*Note*: *n* represents the number of combinations of elements (subjects, postures, cameras, and shots). Protocol A: 10 continuous shots; Protocol B: 3 single shots without picking up the camera; Protocol C: 3 single shots with camera picked up between each shot.

Abbreviations: H, high; L, low; SD, standard deviation.

^a^ We picked the first 3 times of 10 continuous shots.

### 4.2 Variation in skin color measurements between shooting protocols

Regardless of the shooting protocol, the average and variation of the difference from the average showed the same trend, that is, largest in the R color, followed by G, and B. The number of outliers determined as larger than the upper limit or smaller than the lower limit of the 95% CI of the difference from the average was equally distributed among color spaces in the different protocols. Regarding the occurrence frequency of outliers at each shot in protocol A, the most up and low outliers appeared at the 4th and 8th shots, respectively, with the least occurring at the 6 and 7th shots (Table S1).

### 4.3 Variation in skin color measurements between postures

**FIGURE 3:**
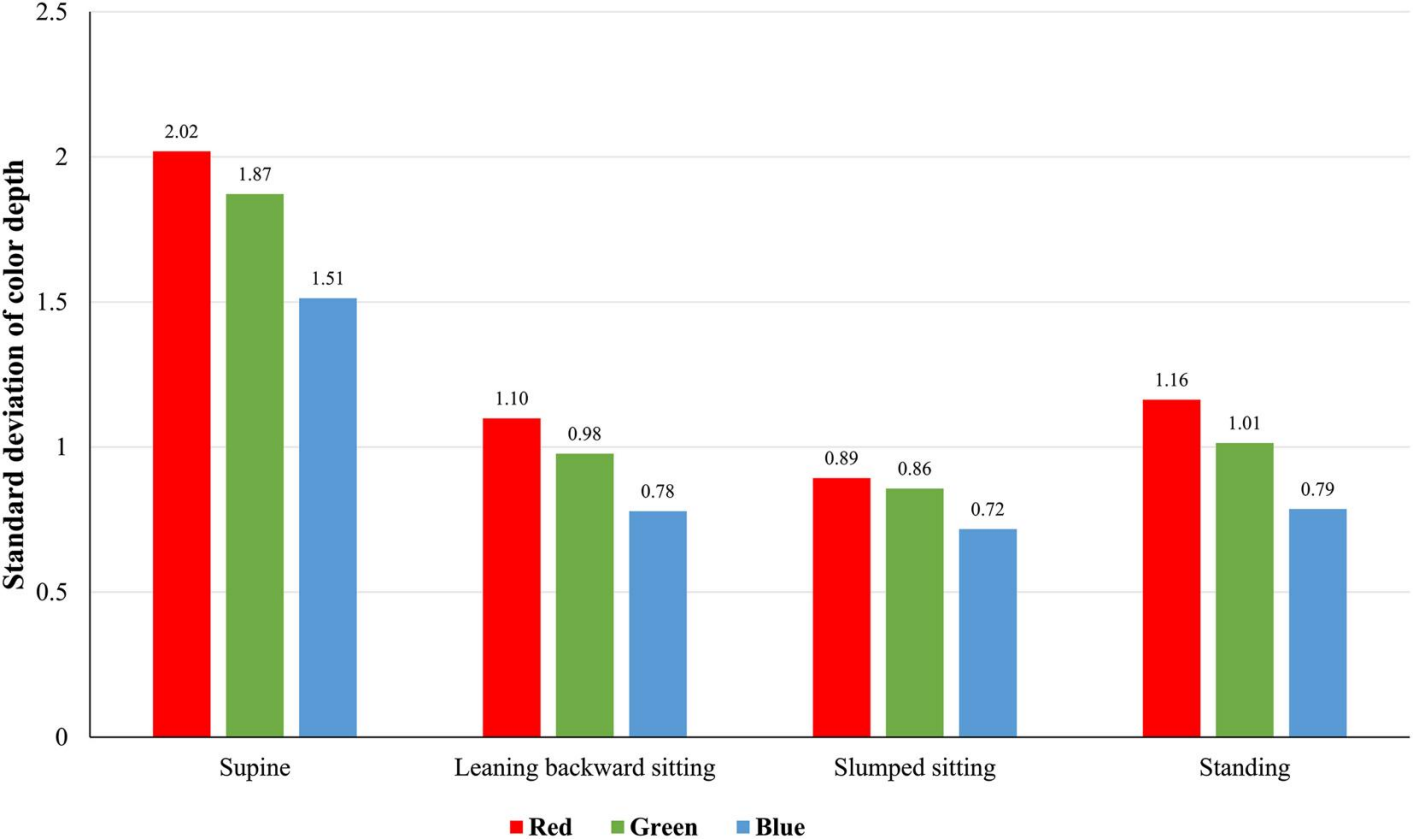
Standard deviations of the difference from the average for the red‐blue‐green colors in different postures

The variations were more obvious between postures than those between shooting protocols (Figure 3). Regardless of posture, blue represents less variation (smallest standard deviation) in color depth compared to the other colors. The standard deviation of differences from the average skin color depth when the subject was in a supine position was nearly two‐fold of the other postures.

### 4.4 The precision of skin color measurements

Interclass correlation coefficient (ICC) and 95% CI of primary color based on the settings (within‐group, camera, protocol, and posture) are shown in Table 2. All replicate skin color measurements were stable under different setting conditions. A perfect ICC (>99%) was observed when looking at measurements at different times (Table 2). The skin color obtained by different cameras reflects lower but remains highly consistent (ICC: 95.3% for red, 92.4% for green, and 93.7% for blue). Large variations may be caused by human factors, with the ICC between postures and protocols reaching 97%.

**TABLE 2:** The interclass correlation coefficient of color depth in different variant sources

	Red			Green			Blue		
Variant source	ICC	95% CI		ICC	95% CI		ICC	95% CI	
Inter group	99.2%	98.9%	99.4%	99.3%	99.1%	99.5%	99.3%	99.1%	99.5%
Camera	95.3%	94.2%	96.2%	92.4%	90.6%	93.9%	93.7%	92.3%	95%
Protocol	98.3%	97.8%	98.7%	98.4%	97.9%	98.8%	98.3%	97.7%	98.8%
Posture	97.8%	97.3%	98.3%	97.9%	97.4%	98.3%	97.6%	96.9%	98.1%

*Note*: Variation sources were from the camera (I, II, III), protocol [(A) 10 continuous shots, (B): single shots without picking up the camera, and (C) 3 single shots with camera picked up between each shot], and posture [(a): supine, (b): leaning backward sitting, (c): slumped sitting, and (d): standing].

Abbreviations: CI, confidence interval; ICC, interclass correlation coefficient.

## 5 DISCUSSION

Skin color is considered one of the critical features for identifying specific skin abnormalities, so modern medicine depends on physician subjective eye observation combined with the patient's symptoms to make clinical decisions. Although skin color measurement by an instrument has potential for clinical applications, only a few studies focus on measuring variations from different sources and consequent implications. The novelty of the current study is the quantification of various sources of uncertainty (subject, protocol, and postures) in human skin color, which should be helpful in future relevant applications.

Skin color changes are mainly detected by the naked eye which lacks objectivity and accuracy. The naked eye can easily discern skin color problems from healthy skin color (either hyperpigmented or hypopigmented), but even experienced physicians find it difficult to grade the severity of skin color disorders. Vitiligo is an autoimmune disorder causing patchy loss of skin pigmentation occurring in 0.76%–1.11% of adults.^12^ Several assessment tools incorporating key determinants of skin lesions have been developed to improve vitiligo disorder treatment.^13^ For example, the Vitiligo Extent Tensity Index score mainly depends on cutaneous and hair pigmentation and the proportion of pigmented and depigmented areas in the total body surface.^14^ Although the assessment method has largely improved uncertainty caused by subjective clinical judgements, applying these methods to identify changes within limited treated skin lesions remains infeasible. With acceptable costs (∼5000 USD), the current device is easy to operate and a non‐invasive approach to accurately and reliably quantify human skin color changes. In addition, applying the developed system to explore the relationship between diseases and skin color in the health field could be helpful. More large‐scale systematic research is expected to determine meaningful skin color changes in human health/disease conditions.

The variability and accuracy of skin color measurement by instruments and algorithms have been explored by previous studies.^15^, ^16^ Wang et al. analyzed the effects of aperture size, pressure, and measurement distance on the variability of facial color detected by a Konica Minolta CM700d spectrophotometer and a PhotoResearch PR650 telespectroradiometer.^15^ Similar to our observation, the differences between measurements for different facial and hand locations were larger than instrument repeatability and the inter‐instrument agreement. The information reflects that more studies exploring skin color variabilities in different body locations are needed. Another study different from our intentions emphasized that not only the stability of one image system but also the image format and skin color characterization algorithm play a key role in determining facial color accuracy.^16^ It is worth noting of even a common commercial facial color detection device named Visia analysis system (Canfield Scientific Inc.) does not transparently offer whether its measurement is standardized to an international color standard. Therefore, a scientific guideline for making skin color instrument development process transparency should be developed.

The experimental results indicate that the 95% CI of the overall difference in color depth of RGB colors are ±2.8, ±2.6, and ±2.1, which are compatible with the accuracy of this device (∆E=3). Although skin color results from the experimental settings were consistently reliable, several novel findings are worthy of note. First, the replicate measurements of 10 continuous shootings were not as stable as those of single shooting approaches, possibly due to the instability of the mechanical camera shutter. In addition, single shooting without picking up the camera provides a smaller variation in measurements than shooting with picking up the camera, reflecting that moving the measurement location may contribute additional variations to repeated skin color measurements. The most inconsistent results were observed in the supine position compared to the other postures. The exact causes are unknown; however, lying down may alleviate abdominal tension, increase diaphragmatic breathing, and add measurement variability. Finally, the lowest ICC among our experiments was tested between different cameras, which suggested a single camera for one study may be needed if skin color changes are the main research interest.

Some color spaces are commonly applied for detecting color by devices. Our developed devices adopted the RGB rather than HSV or L*A*B color space, which is more instinctive and straightforward for medical professionals to apply. Although HSV and L*A*B color spaces are easily calculated, a recent study showed a weak correlation between the *L* value and human perception of skin color, which may limit its application in skin color detection.^17^ Several potential skincare applications of this device should be emphasized. First, the traditional Fitzprick skin type may lose its accuracy in distinguishing the borderline skin type between adjacent categories. The device undoubtedly could categorize human skin type on a tremendously detailed scale. Then, in a similar previous study, the device could trace the progression and the response of vitiligo and its therapies.^18^ Third, it could detect flap failure by measuring the color of the flap to determine whether the flap was successful after plastic surgery. Finally, it could assist a physician in determining the progression of jaundice, so they are no longer dependent on periodic blood tests.^19^ More studies exploring its feasibility and accuracy in different healthcare domains are warranted.

This study has some limitations. First, the study only included Asian subjects located in a subtropic area, so the results may not be generalizable to other uncollected variances such as environmental factors. Although the reliability of the system was not tested in extreme environmental conditions (temperature, humidity…, et al.), it should not affect the standard applications in most usual conditions. All study subjects were healthy and had little hair, so the reliability of our device is unknown when applying it to participants with much hair and intense skin color such as jaundice. In addition, there are numerous skin color‐changing diseases caused by different pigmentation disorders. Sensitivity of the device in determining each pigmentation disease needed to be further tested. Finally, the interoperator variances in skin color measurements were not evaluated as previously,^20^ however, this variance is usually controlled by an operator training programme before initiating a formal experiment.

## 6 CONCLUSION

In conclusion, this study demonstrated that the developed device could stably and reliably detect human skin color across different sources of variation including operating protocols, and respondents' postures. Furthermore, this device has the potential to be used to explore the relationships between health/disease and skin color changes.

## CONFLICT OF INTEREST

The authors declare that there is no conflict of interest that could be perceived as prejudicing the impartiality of the research reported.

## AUTHOR CONTRIBUTIONS

**Chien‐Chung Chen**: Data curation (equal); Formal analysis (equal); Methodology (equal); Visualization (lead); Writing – original draft (lead). **Cheng‐Yin Chung**: Writing – original draft (equal). **Yi‐Wen Chiu**: Conceptualization (equal); Methodology (lead); Supervision (equal); Writing – review & editing (equal). **Yu‐Hsuan Lin**: Funding acquisition (equal); Methodology (equal); Writing – review & editing (equal). **Ling‐Shan Tse**: Resources (equal); Writing – review & editing (equal). **Ching‐Ying Wu**: Writing – original draft (equal); Writing – review & editing (equal). **Shang‐Jyh Hwang**: Resources (equal); Writing – review & editing; (equal). **Ming‐Yen Lin**: Data curation (lead); Formal analysis (lead); Methodology (lead); Writing – original draft (equal); Writing – review & editing (lead).

## ETHICS STATEMENT

All of the study procedures were approved by the Institutional Review Board of Kaohsiung Medical University Hospital (KMUHIRBEXEMPT(I)‐20170144).

## Supporting information

Supporting Information S1

Supporting Information S2

Supporting Information S3

Supporting Information S4

## Data Availability

The data that support the findings of this study are available from the corresponding author upon reasonable request.
