# Supplementary material for: Reliability analysis of a novel measurement system for quantifying human skin color
Source: Skin Health Dis. 2022 Oct 17;3(1):e182. doi: 10.1002/ski2.182 (PMC9892441; doi:10.1002/ski2.182)
Supplement: Supplementary file 1 — Supporting Information S1 [file SKI2-3-e182-s002.docx]

# Table S1. The number of color space outliers detected in sequent ten continuous shots of human skin

| Color space |  | Sequent ten continuous shots | | | | | | | | | | Sum |
| --- | --- | --- | --- | --- | --- | --- | --- | --- | --- | --- | --- | --- |
|  |  | I | II | III | IV | V | VI | VII | VIII | IX | X |  |
| Red | Up | 1 | 1 | 0 | 3 | 2 | 0 | 0 | 1 | 1 | 1 | 10 |
|  | Low | 2 | 0 | 1 | 1 | 1 | 0 | 0 | 3 | 2 | 0 | 10 |
|  | Total | 3 | 1 | 1 | 4 | 3 | 0 | 0 | 4 | 3 | 1 | 20 |
| Green | Up | 1 | 2 | 0 | 4 | 2 | 1 | 0 | 2 | 2 | 1 | 15 |
|  | Low | 2 | 1 | 1 | 1 | 1 | 0 | 1 | 4 | 2 | 1 | 14 |
|  | Total | 3 | 3 | 1 | 5 | 3 | 1 | 1 | 6 | 4 | 2 | 29 |
| Blue | Up | 2 | 2 | 0 | 4 | 2 | 0 | 0 | 1 | 2 | 1 | 14 |
|  | Low | 2 | 1 | 1 | 1 | 1 | 0 | 0 | 3 | 1 | 1 | 11 |
|  | Total | 4 | 3 | 1 | 5 | 3 | 0 | 0 | 4 | 3 | 2 | 25 |
| Overall | Up | 4 | 5 | 0 | 11 | 6 | 1 | 0 | 4 | 5 | 3 | 39 |
|  | Low | 6 | 2 | 3 | 3 | 3 | 0 | 1 | 10 | 5 | 2 | 35 |
|  | Total | 10 | 7 | 3 | 14 | 9 | 1 | 1 | 14 | 10 | 5 | 74 |

The number of outliers determined as larger than the upper limit or smaller than the lower limit of the 95% confidence interval of the difference from the average.

Table S2. The standard deviation of difference from the average in study subjects, by red-blue-green colors and posture

|  |  | Subject | | | | Overall |
| --- | --- | --- | --- | --- | --- | --- |
|  |  | 1 | 2 | 3 | 4 |  |
| Red | Supine | 1.48 | 1.89 | 2.71 | 1.86 | 2.02 |
|  | Leaning backward sitting | 1.18 | 1.36 | 0.86 | 0.97 | 1.10 |
|  | Slumped sitting | 0.73 | 0.82 | 1.07 | 0.94 | 0.89 |
|  | Standing | 1.09 | 0.90 | 1.65 | 0.88 | 1.13 |
|  |  |  |  |  |  |  |
| Green | Supine | 1.25 | 1.72 | 2.35 | 2.06 | 1.87 |
|  | Leaning backward sitting | 1.02 | 1.17 | 0.77 | 0.93 | 0.98 |
|  | Slumped sitting | 0.73 | 0.74 | 0.97 | 0.99 | 0.86 |
|  | Standing | 0.98 | 0.78 | 1.38 | 0.84 | 1.01 |
|  |  |  |  |  |  |  |
| Blue | Supine | 0.92 | 1.56 | 1.86 | 1.60 | 1.51 |
|  | Leaning backward sitting | 0.71 | 1.05 | 0.61 | 0.70 | 0.78 |
|  | Slumped sitting | 0.56 | 0.68 | 0.79 | 0.83 | 0.72 |
|  | Standing | 0.71 | 0.65 | 1.07 | 0.67 | 0.79 |

# Supplement figure legend

Figure S1. Bland-Altman plots for repeated a)red, b) blue, and c) green measurements by different protocols in the study subjects.
