# Supplementary figures and images for: Reliability analysis of a novel measurement system for quantifying human skin color
Source: Skin Health Dis. 2022 Oct 17;3(1):e182. doi: 10.1002/ski2.182 (PMC9892441; doi:10.1002/ski2.182)

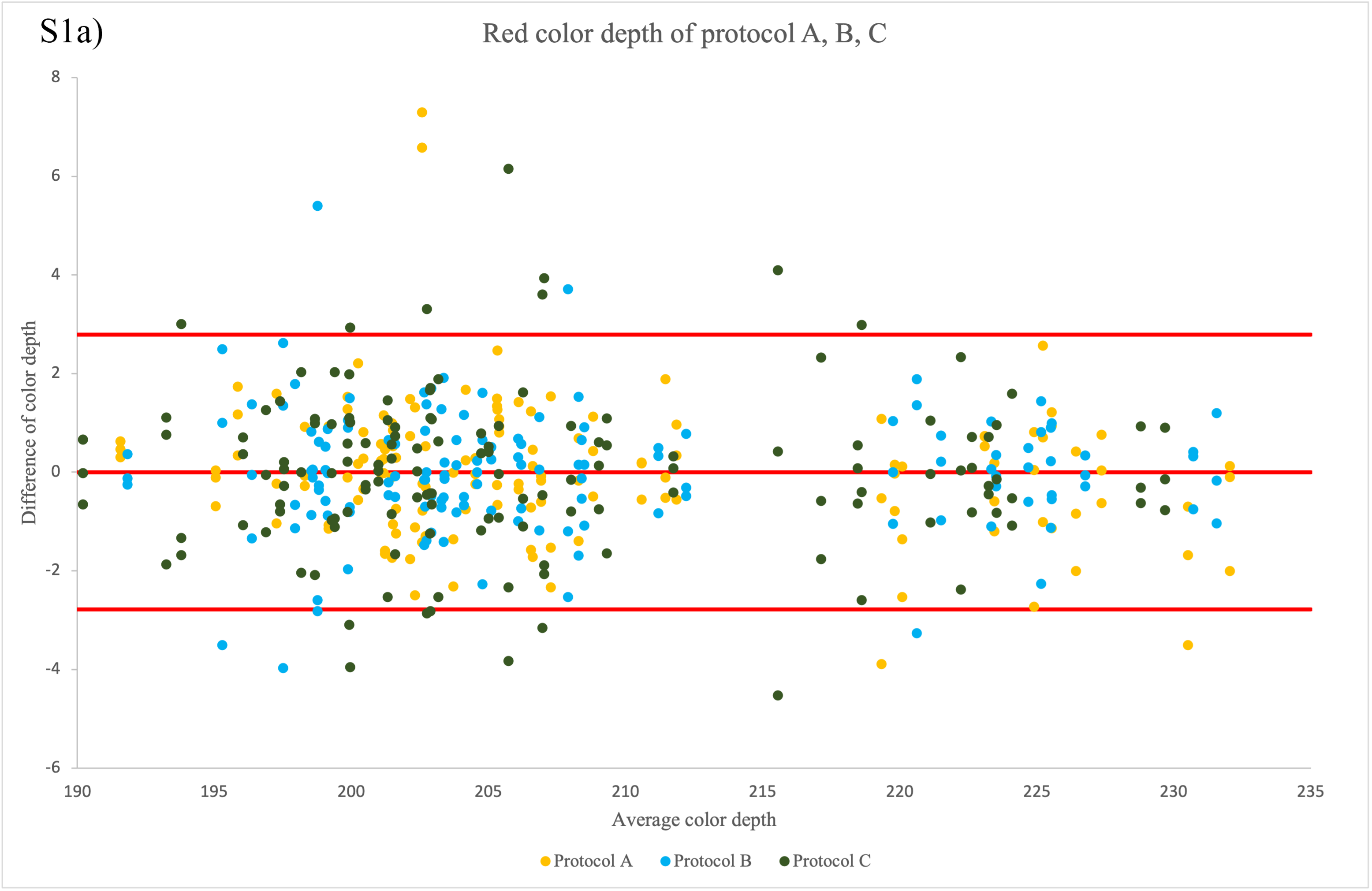

Supplement: Supplementary file 2 — Supporting Information S2 [file SKI2-3-e182-s004.png]

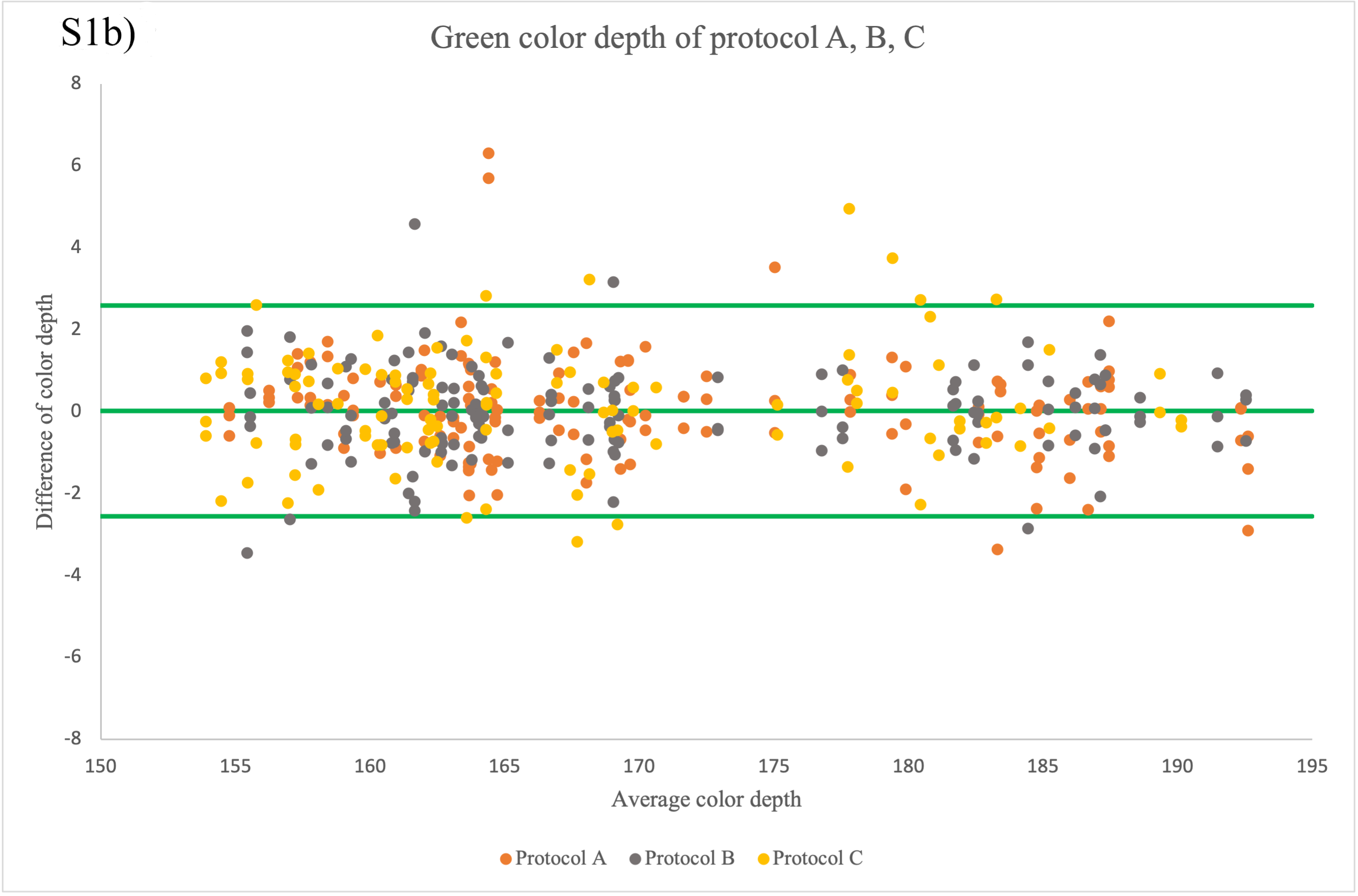

Supplement: Supplementary file 3 — Supporting Information S3 [file SKI2-3-e182-s001.png]

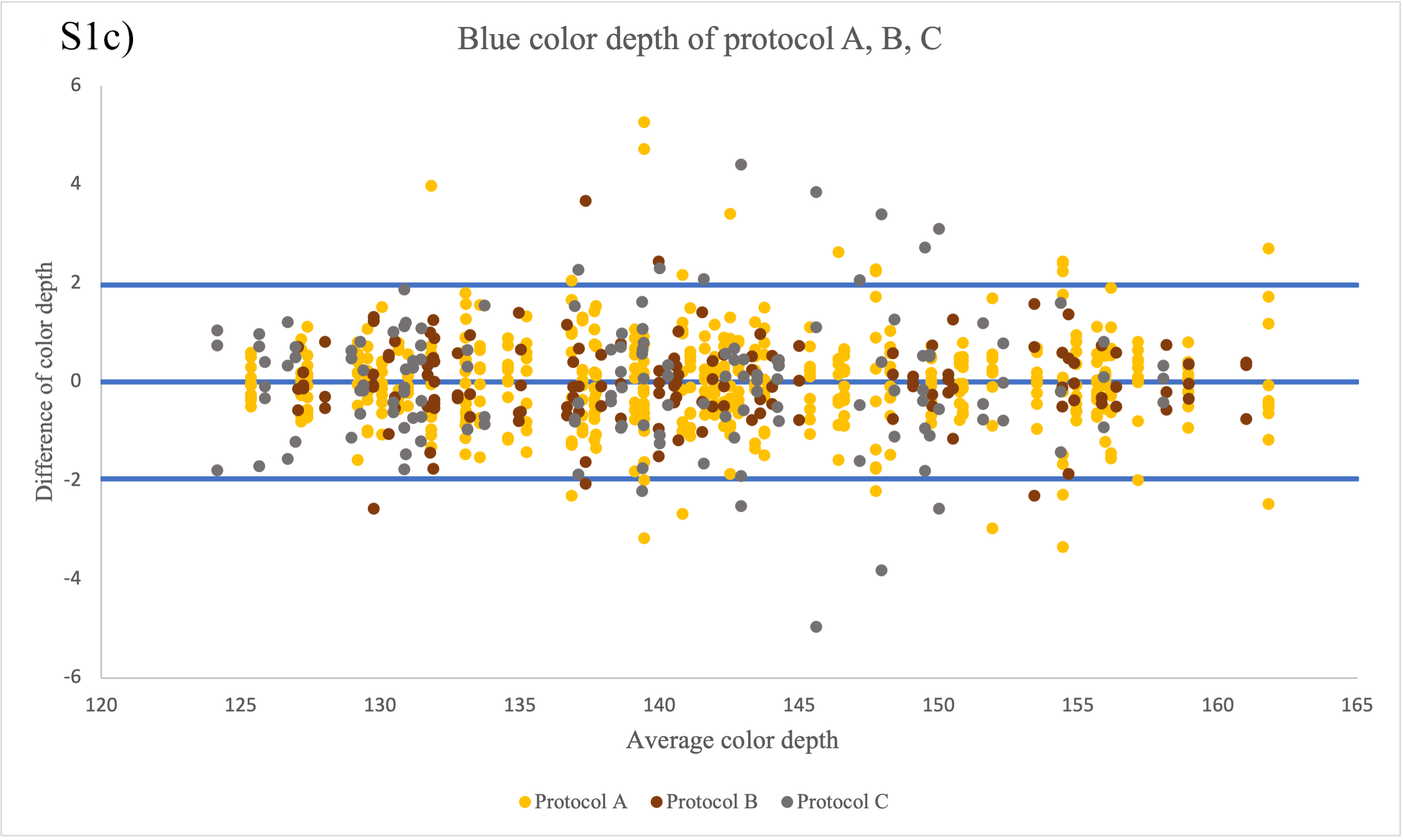

Supplement: Supplementary file 4 — Supporting Information S4 [file SKI2-3-e182-s003.png]
